# Antigenicity and Safety Evaluation of *Lactiplantibacillus plantarum* 7-2 Screened to Reduce α-Casein Antigen

**DOI:** 10.3390/foods11010088

**Published:** 2021-12-29

**Authors:** Guangqing Mu, Zhao Zhang, Jiayi Wang, Shujuan Jiang, Hongxin Wang, Yunpeng Xu, Xinling Li, Lei Chi, Yue Li, Yanfeng Tuo, Xuemei Zhu

**Affiliations:** 1 School of Food Science and Technology, Dalian Polytechnic University, Dalian 116034, China; guangqingmu@163.com (G.M.); Zhangzhao5337@163.com (Z.Z.); wangjiayittkx@126.com (J.W.); jianglaoda2005@126.com (S.J.); 18314113036@139.com (H.W.); xyp.giveup@163.com (Y.X.); tyfjq@aliyun.com (Y.T.); 2 Xinjiang Tianrun Biological Technology Co., Ltd., Urumchi 830011, China; xjlxl911@163.com; 3 Dalian Women and Children Medical Center, Dalian 116012, China; chilei00070@163.com (L.C.); ertongyufang@163.com (Y.L.)

**Keywords:** milk protein allergy, α-CN, LAB fermentation, *Lactiplantibacillus plantarum*

## Abstract

α-Casein (α-CN) is considered the main allergen in bovine milk. Lactic acid bacteria (LAB) fermentation can hydrolyze milk protein and therefore reduce the antigenicity. In this paper, a LAB reducing the antigenicity of casein, identified as *Lactiplantibacillus* *Plantarum* 7-2 (*L. plantarum* 7-2), was primarily identified by screening for protein hydrolysis ability using a method involving the determination of released free amino acid with further selection for the ideal antigenicity-reducing capability by enzyme-linked immunosorbent assay (ELISA). In order to verify the capability of *L. plantarum* 7-2 in inhibiting antigenicity, the standard milk proteins α-LA, β-LG, α-CN, β-CN and κ-CN were cultured with *L. plantarum* 7-2 for 18 h; The results of SDS-PAGE show that all the bands corresponding to the full length tested proteins became unclear or completely disappeared indicating that these proteins were hydrolyzed by *L. plantarum* 7-2. Correspondingly, the antigenicities of α-CN and β-LG were significantly reduced. *L. plantarum* 7-2 demonstrated negative hemolysis and nitrate reductase capabilities and was sensitive to the commonly used antibiotics ampicillin clindamycin tetracycline chloramphenicol, and erythromycin, demonstrating that *L. plantarum* 7-2 could be used in dairy product fermentation to reduce the antigenicity of milk protein.

## 1. Introduction

Statistics from the World Allergy Organization indicate that 220–520 million people worldwide suffer from food allergies [1]. In recent years, allergic diseases have attracted increasing attention, and studies have shown that allergies and adverse reactions to food ingredients are becoming more common [2]. Food-based allergic reactions are mainly caused by eight kinds of protein-rich foods, including milk, eggs, fish, crustaceans, shellfish, peanuts, soybeans, nuts, and wheat [3]. The distribution of food allergy diseases depends on the population, diet, and reference standards. Milk protein allergies are becoming more common in feeding infants and young children, affecting around 3–5% of this population. This allergenicity is a very vital issue because milk is among the main sources of nutrition for infants and young children [4]. The exogenous amino acids provided by milk protein play an important role in the growth and development of organs and ensure the normal function of newborns. A severe milk allergy will affect the function of organs and systems and may even cause anaphylactic shock in extreme cases [5]. The common symptoms caused by milk protein allergy are skin lesions, respiratory tract lesions and intestinal lesions. In addition, milk protein allergies can lead to the destruction of intestinal villi, which leads to the lack of lactase and lactose intolerance. Complications of milk protein allergies may also include nutritional and growth impairment, anemia and insufficient bone calcification [6].

The main protein components of milk include casein (α_s1_, α_s2_, β and κ-CN) and whey proteins (β-lactoglobulin, α-whey protein, lactoferrin and immunoglobulin). Milk contains more than 30 proteins with potential allergic properties [7]. Casein content accounts for about 80% of milk protein content, of which α-CN (including α_s1_ and α_s2_-CN) accounts for about 80% of casein content. As a non-existent part of human milk, α-CN is most likely to cause allergies. At least five antigenic determinants have been identified in various parts of the protein sequence: αs1, 28–50aa, 75% reactive and 26.7% tolerant; αs2, 1–20aa, 75% reactive and 13.3% tolerant; 13–32aa, 75% reactive and 26.7% tolerant; 67–86aa, 75% reactive and 33.3% tolerant; and 181–207aa, 75% reactive and 20% tolerant [8,9]. β-Lactoglobulin (β-LG) is characterized by high resistance to enzymatic hydrolysis. In the immature digestive tract of infants, β-LG may lead to the transfer of unhydrolyzed protein antigens to the blood and induce an acute immune response [10].

At present, protein allergenicity can be reduced through physical methods (heating, irradiation, ultra-high pressure, etc.) that change the spatial structure or through biochemical methods (enzyme hydrolysis, microbial metabolism, reagent induction) that alter the amino acid residues or eliminate allergens via the polypeptide hydrolysis of proteins [11,12]. Byun et al. found that irradiating shrimp results in a 70% decrease in IgE binding capacity [13]. Kleber showed that the antigenicity of skim milk increased and reached its highest point at 90 °C and then decreased with the increase in temperature [14]. Penas et al. demonstrated that protease treatment could reduce the sensitization of soybean whey protein under high pressure (100~300 MPa) for 15 min [15]. Pintado and Malcata reported that the hydrolytic effect differed depending on the protease [16]. The hydrolytic effect of pepsin on α-lactalbumin (α-LA) was higher than that of trypsin, while the hydrolytic effect of trypsin on (β-LG) was stronger. Kim et al. found that pepsin and trypsin could reduce the sensitization of heat-treated whey protein [17]. Hussein et al. reported that the immunoreactivity of buffalo milk protein hydrolysates and sera of patients with milk allergy was reduced to 30% and 16% by pepsin hydrolysis, respectively, indicating that buffalo milk proteins treated with pepsin had significantly decreased allergenicity [18]. According to the above research, we found that the physical methods used to alter protein allergenicity worked mainly by changing the protein spatial structure to eliminate the conformation of allergic epitopes, while more linear allergic epitopes may be exposed and, thus, allergenicity would be increased by physical treatment. Compared with physical methods, protein can be hydrolyzed by microbial enzymes to destroy allergic epitopes, so it is important to choose appropriate methods to effectively reduce or eliminate casein allergens.

Fermentation is a traditional processing technology in the food industry, and fermented food has a positive effect on human health. One of the reasons for this is that microbial enzyme-hydrolyzed protein releases bioactive peptides during fermentation. Studies have shown that lactic acid bacteria (LAB) have a complex proteolysis system composed of protease (extracellular protease), peptidase and transport system. LAB are used as a starter for preparing fermented dairy products and they also play an important role in the regulation of allergy. At present, LAB fermentation is one of the more effective ways to reduce the allergy to milk proteins. On the one hand, LAB fermentation can improve the bioavailability of milk protein; on the other hand, milk protein will be degraded into small molecules of peptides and amino acids after fermentation. This will destroy the allergic epitopes in milk proteins, thus reducing their allergenicity. Studies have shown that, as a method to reduce the allergenicity of milk protein, LAB fermentation decreases the allergenicity of milk protein in the substances secreted in microbial metabolisms, such as acidic metabolites and extracellular polysaccharides. Lactic acid and γ-aminobutyric acid may affect the secondary and tertiary protein structure, altering epitopes and thus reducing allergenicity [19]. Moreover, it has been proven that fermentation can degrade some food allergens. The antigenicity of whey protein decreased significantly after fermentation by LAB with an inhibition rate of 53–87% for α-LA and 86–95% for β-LG [20]. Fotschki et al. found that in horse cheese protein degraded by fermentation with *Lactobacillus*, *Streptococcus thermophilus* and *Bifidobacterium,* the antigenicity of β-CN decreased by 74% [21]. Additionally, 85% of β-CN and 68% of α-CN were hydrolyzed by fermentation with *Lactobacillus fermentum* IF3956; correspondingly, the antigenicity of α-CN was effectively reduced [22,23]. Ying et al. determined that *Lactobacillus German* combined with pepsin hydrolysis reduced of antigenicity of β-LG by 5% [24]. Wroblewska et al. showed that the remaining antigenicity of whey protein in fermented milk only remained 1% [25]. According to the above studies, it was concluded that fermentation has obvious advantages in degrading allergenic proteins [26,27].

The objective of this paper was to screen LAB for their ability to reduce milk protein allergenicity in fermented milk from Xinjiang, China. First, 56 strains of LAB were screened for their ability to reduce the antigenicity of α-CN. Then, the ability of the final screened LAB to reduce milk protein allergenicity was tested by incubation with α-CN, β-CN, α-LA and β-LG.

## 2. Materials and Methods

### 2.1. Strains and Materials

Fifty-six strains were isolated from traditional fermented dairy products in Xinjiang and preserved in Dalian key Laboratory of Functional Probiotic and Protein.

The skim milk powder was provided by Beijing Sanyuan Food Limited Company China. The powder contained dry matter 96.2%, total proteins 32.9% and carbohydrates 54.5%.

Enzyme-linked immunosorbent assay (ELISA) kits (ELISA Kit for α-CN, ELISA Kit for β-CN, ELISA Kit for α-LA and ELISA Kit for β-LG) were obtained from CLOUD-CLONE Corp., Wuhan, China.

The antigen proteins used for sensitization studies and ELISA were α-LA from bovine milk (L6010; purity 85%; lyophilized power), β-LG from bovine milk (L3908; purity, 90%; containing β-LG A and B; lyophilized power), α-CN from bovine milk (C6780; purity 70%; lyophilized power), β-CN from bovine milk (C6905; purity 98%; lyophilized power) purchased from Merck KGaA, Darmstadt, Germany.

Protein Quantification Kit (BCA kit) purchased from Merck KGaA, Darmstadt, Germany.

### 2.2. Reconstituted Milk

Skim milk powder was prepared as an emulsion with a content of 11% (*w*/*w*), and then sterilized at 105 °C for 15 min [28]. After heat treatment, the milk was allowed to naturally cool to room temperature in a sterile environment.

### 2.3. Screening and Identification of LAB with Reducing α-CN

The selected 56 strains were incubated at 37 °C and subcultured in the same MRS medium for two or three generations for their activation. MRS medium: glucose 20 g, peptone 10 g, Beef extract 10 g, yeast extract 5 g, ammonium citrate dibasic 2 g, K_2_PO_4_ 2 g, CH_3_COONa 5 g, MgSO_4_ 0.58 g, MnSO_4_ 0.25 g, TWEEN-80 1 mL, H_2_O 1000 mL, sterilized at 121 °C for 20 min.

Then, the 56 strains of bacteria were further cultured with inoculation of 4% on 11% skim milk for fermentation at 37 °C for 24 h. The content of free amino acids in fermented milk was determined using the Church [29] o-phthalaldehyde (OPA) method. In detail, 5 mL samples were mixed with 1 mL H_2_O and 10 mL trichloroacetic acid and centrifuged for 5 min at 3000× *g* rpm. To The supernatant was added 3 mL OPA reagent (50 mL 0.1 mM sodium borate, 5 mL 20% sodium dodecyl sulfate (SDS), 80 mg phthalaldehyde, 2 mL CH_3_OH, 200 μL DMSO, and 200 mL H_2_O). After two minutes, the absorbance value at 340 nm was measured at 25 °C. The equation for tyrosine content based on the standard curve is:

Y = 1.2162X + 0.0262 (R^2^ = 0.9916) (1)

Determination of α-CN antigenicity: The strains producing higher amounts of free amino acid were selected and fermented in a liquid medium containing 11% (*w*/*w*) skim milk with 4% bacteria at 37 °C for 24 h. The content of α-CN antigen was detected using an ELISA kit. Centrifuge the sample under 3500 rpm for 5 min to obtain the supernatant. First, the sample supernatant was added to a 96-well plate, 100 μL per well, and standing at 37 °C for 1 h. Then the liquid in the well was discarded and the 100 μL detection solution A (Biotinylated β-CN antibody) was placed at 37 °C for 1 h in each well, and the wells were washed with eluent 3 times. Next, the 100 μL detection solution B (HRP labeled avidin) was added to each well with incubation at 37 °C for 1 h, and the wells were then washed with eluent 5 times. Then 90 μL TMB substrate was added to each well followed by incubation at 37 °C for 15 min. Finally, 50 μL of the terminator (2 mol/L H_2_SO_4_) were added and the plate was immediately read at 450 nm. The casein antigen concentration reduction rate was calculated using the following equation:

C = (A0 − A1)/A0 × 100%(2)

C = casein antigen concentration reduction rate(%); A_0_ = antigen concentration of unfermented skim milk; A_1_ = sample antigen concentration.

### 2.4. Strain Safety

#### 2.4.1. Hemolysis Characteristics

The *L. plantarum* 7-2 was cultured in a sheep blood agar medium and the hemolysis characteristics of the strain were determined using *Listeria monocytogenes* as a positive control.

#### 2.4.2. Nitrate Reductase Test

A nitrate reductase test was used to determine the ability of the strain to produce nitrate reductase. The tested strains were inoculated in a broth medium containing nitrate and cultured for 6 h. Following centrifugation, the supernatant was collected and a saturated potassium iodide solution was added, followed by shaking, and a starch indicator was then added to allow observation of the subsequent color reaction.

#### 2.4.3. Drug Sensitivity

Ampicillin, gentamicin, erythromycin, clindamycin, tetracycline, chloramphenicol, and vancomycin were the antibiotics selected for testing the drug resistance of the strains according to the drug sensitivity paper method [30].

#### 2.4.4. Biogenic Amines

Biogenic amines analysis was conducted using HPLC as described by Liu et al. [31]. Chromatographic conditions: Waters e2695 HPLC, PDA detector 2998, chromatographic column (C18 4.6 × 250 mm, 5 μm), UV detection wavelength 254 nm, column temperature 30 °C, phase A: ultra-pure water, mobile phase B: acetonitrile, flow rate is 1.0 mL/min, injection volume is 20 μL, gradient elution.

### 2.5. Identification of Strain

The colony morphology of the final selected strains was observed by a Gram staining test after 48 h of anaerobic incubation at 37 °C on MRS agar plates. The total DNA of stains was extracted using a DNA extraction kit (Axygen, CA, USA) for biomolecular identification. The PCR amplifications were carried out as follows: 3 min at 94 °C; 94 °C for 30 s 54 °C for 30 s, and 72 °C for 45 s, cyclic amplification 30 times; final extension at 72 °C for 10 min [32]. The sequence was searched and compared to the nucleotide database from the National Center for Biotechnology Information (NCBI) GeneBank using the BLAST program. A phylogenetic tree based on the 16S rRNA gene sequences of the ideal isolates considered in this study was constructed using MEGA 7.0.

### 2.6. Optimization of Fermentation Conditions

#### 2.6.1. Inoculation Amounts

The *L. plantarum* 7-2 was inoculated in 11% skim milk according to different inoculation amounts (2%, 4%, 6%, 8%) and fermented at 37 °C for 24 h. The content of α-CN antigen and the number of live LAB were determined under different inoculum.

#### 2.6.2. Fermentation Time

After the inoculation amount was determined, the target strain was inoculated in 11% skim milk according to different fermentation times (6 h, 12 h, 18 h, 24 h) and fermented at 37 °C. The content of α-CN antigen and the number of living LAB were determined at different inoculation times.

#### 2.6.3. Culture Temperatures

The amount of inoculation and fermentation time were determined and the target strains were inoculated in 11% skim milk according to different culture temperatures (25 °C, 31 °C, 37 °C, 42 °C). The content of α-CN antigen and the number of living LAB were determined at different inoculation times.

Test method for the number of living LAB: the plate count was determined by the 10-fold dilution method. The gradient dilution was carried out with the concentration of 8% normal saline, and the suitable three gradients were selected and diluted in MRS plate solid medium at 37 °C for 48 h.

### 2.7. Verification of Antigen Reducing in Fermented Standard Proteins and Pasteurized Milk

#### 2.7.1. Standard Milk Protein Medium Fermentation and Sample Preparation

*L. plantarum* 7-2 with 4% inoculum was fermented with different milk proteins media. According to the content of milk protein in milk, the concentrations of standard milk proteins were: α-LA (1.6 mg/mL), β-LG (4.0 mg/mL), α-CN (15.36 mg/mL) and β-CN (12.48 mg/mL). After incubation at 37 °C for 18 h, the supernatant was obtained by centrifugation at 3500× *g* rpm for 5 min. The protein concentration of the supernatant was determined by the BCA kit.

#### 2.7.2. Antigenicity Test

The antigenicity of four milk proteins was determined according to the description of α-CN antigenicity detection ELISA method in 2.4. The fermentation of pasteurized milk was also determined as a reference control. *L. plantarum* 7-2 was inoculated in a sterile environment and fermented in pasteurized milk at 37 °C for 18 h with an inoculation amount of 4%.

#### 2.7.3. SDS-PAGE

Adjust the protein concentration in the supernatant of each milk protein to 1.6 mg/mL. Under reduction conditions, SDS-PAGE electrophoresis of the above samples was carried out using a 15% (*w*/*w*) separation gel and 5% (*w*/*w*) concentrated stacking gel. Each sample was mixed with the buffer solution (in the presence of 5% β-mercaptoethanol) at 1:1 and heated for about 5 min at 100 °C, then loaded into different slots. Each slot 10μL. Following sample addition, constant current (concentrated gel 16 mA, separation gel 32 mA) electrophoresis was carried out. After electrophoresis, the gel was stained with Coomassie Brilliant Blue R250 for 4–5 h, then decolorized in decolorizing solution (C_2_H_5_OH 500 mL, CH_3_COOH 90 mL, H_2_O 410 mL) until the background was clear and then scanned on a gel imager (C300 Azure Biosystems, Dublin, CA, USA) [33].

### 2.8. Statistical Analysis

All the experiments were repeated three times. The statistical analysis of the data was carried out using SPSS 9.0 statistical software. The significance of the dates was assessed by Duncan’s multiple-range test and the significant level was 0.05.

## 3. Results and Discussion

### 3.1. Strain Screening

**Figure 1:**
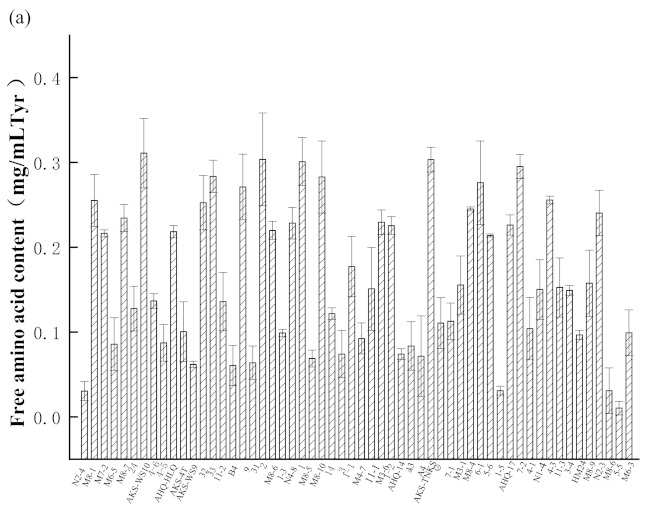

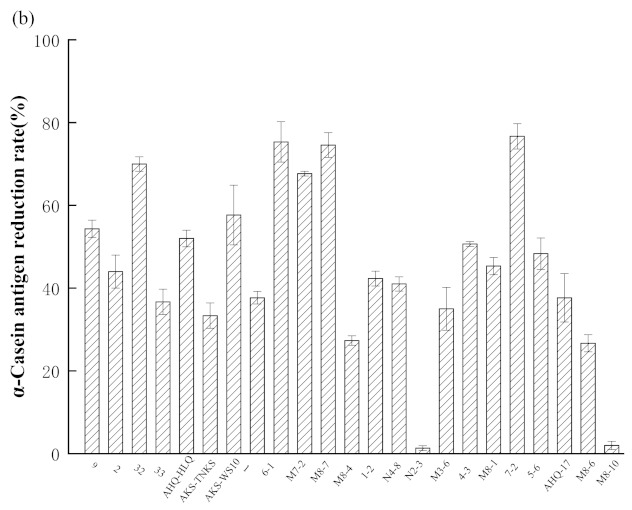
(**a**) Free amino acid content of skim milk fermented by 56 strains of bacteria. (**b**) Antigenic changes of α-casein in 23 strains of bacteria.

A strain capable of reducing the antigenicity of casein was selected from 56 strains of Xinjiang in China by determining the free amino acid content and casein antigen in fermented skim milk. First, the free amino acids of 56 strains of fermented milk were determined (Figure 1a). There were 23 strains, in which the level of free amino acid was higher than 0.2 mg/mL. With sufficient nutrition in the fermentation stage, strains grew rapidly and possessed strong reproduction ability. Some LAB produced extracellular proteases, which, to some extent, destroy the allergic epitopes of milk protein and reduce the allergenicity of milk protein [19]. Additionally, the content of free amino acids explained the ability of LAB to hydrolyze milk protein and the extracellular protease activity of LAB was inferred.

**Figure 2:**
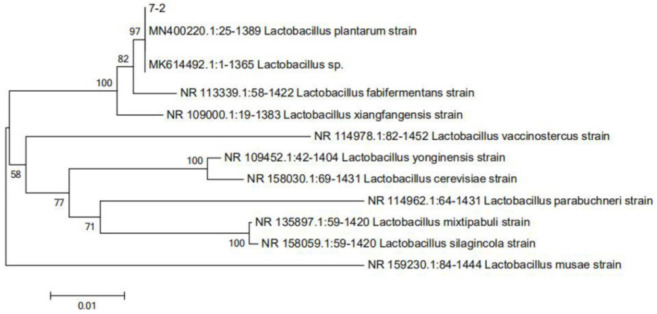
Phylogenetic tree of strain 7-2.

The strain with the best antigen changes rate was analyzed through the difference of α-CN antigenicity of fermented milk (Figure 1b). The determination of antigenicity by ELISA is proven to be a feasible and effective method. Wu et al. showed that the antigenicity of β-lactoglobulin decreased significantly after the Maillard reaction, whereby ELISA was used to determine the binding of β-lactoglobulin to two kinds of immunoglobulin before and after the Maillard reaction [34]. Jedrychowski et al. demonstrated that the antigenicity of fermented milk with different heat-sensitive strains and the antigenicity detected by ELISA was lower than that of raw milk [35]. In this case, strains of 7-2, M8-7 (*L. plantarum*) and 6-1 (*L.* *fermentum*) showed a better capacity for reducing the content of α-CN antigen than other strains. According to the above reports, we speculate that these three strains could effectively reduce the antigenicity of α-CN. Combined with the results determined for free amino acid content, strain 7-2 had hydrolyzed the highest amount of milk protein of the tested strains, resulting in the greatest reduction in antigenicity. Therefore, it was selected as the target strain, and based on the 16S rRNA sequence, was determined to be *Lactobacillus plantarum* (Figure 2) and named *Lactobacillus plantarum* 7-2 (*L. plantarum* 7-2).

### 3.2. Safety Evaluation

**Figure 3:**
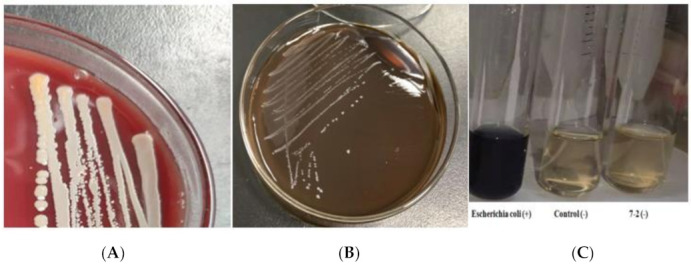
Hemolysis and nitrate reductase test. Picture (**A**) shows the hemolysis positive control and picture (**B**) shows the hemolysis of *L. plantarum* 7-2. Picture (**C**) shows nitrate reductase test.

An excessive intake of biogenic amines may cause headaches, poisoning and other serious or even life-threatening health problems. Among them, histamine is known to be the most toxic biological amine, while putrescine and cadaverine are not only precursors of carcinogen nitrosamine but are also capable of enhancing the toxicity of histamine by inhibiting histamine degrading enzymes. Nitrate reductase is an enzyme that can reduce the nitro group to the amine. The result of the nitrate reductase test showed that *L. plantarum* 7-2 was negative, indicating that *L. plantarum* 7-2 could not produce nitrate reductase (Figure 3). Putrescine was not detected by examining biogenic amines during fermentation with *L. plantarum* 7-2, which contained tryptamine at 4.2345 mg/kg, cadaverine at 2.4258 mg/kg, histamine at 0.5827 mg/kg, tyramine at 2.9750 mg/kg and spermidine at 3.7835 mg/kg (Table 1). All the biogenic amine contents were within the safe range. Furthermore, it was safe in the aspect of hemolytically, on the basis of the negative hemolysis reaction as determined from observation when incubating *L. plantarum* 7-2 on sheep blood agar (Figure 3).

Antibiotic sensitivity should be considered an important index to evaluate safety because different kinds of LAB are widely resistant to antibiotics, leading to potential food safety problems [36]. Seven antibiotics ampicillin, gentamicin, erythromycin, clindamycin, tetracycline, chloramphenicol, and vancomycin were selected for a drug resistance test of *L. plantarum* 7-2 (Table 2). According to the different bacteriostatic zones of *L. plantarum* 7-2 to different antibiotics, it was found that *L. plantarum* 7-2 was highly sensitive to ampicillin, clindamycin, tetracycline, erythromycin, and chloramphenicol, and insensitive to gentamicin and vancomycin. Different strains of LAB have different responses to antibiotics and most of them are sensitive or semi-resistant, and the sensitivity of LAB strains to different antibiotics of the same kind of drugs also differed widely [37]. It was thus determined that it would be feasible for *L. plantarum* 7-2 to be used in food fermentation.

### 3.3. Optimization of Fermentation Condition for Reducing α-CN Antigen

**Figure 4:**
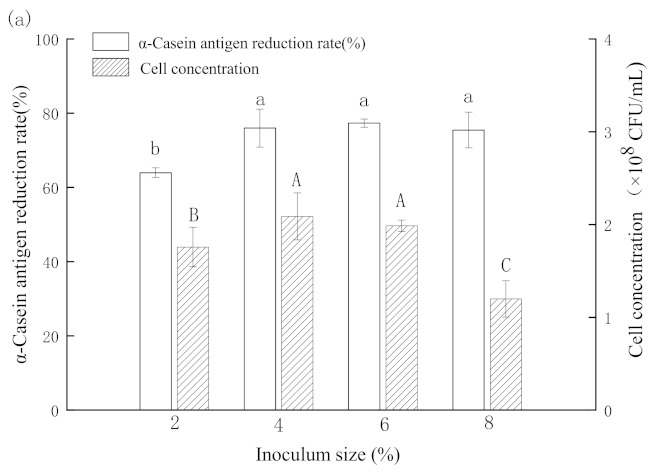

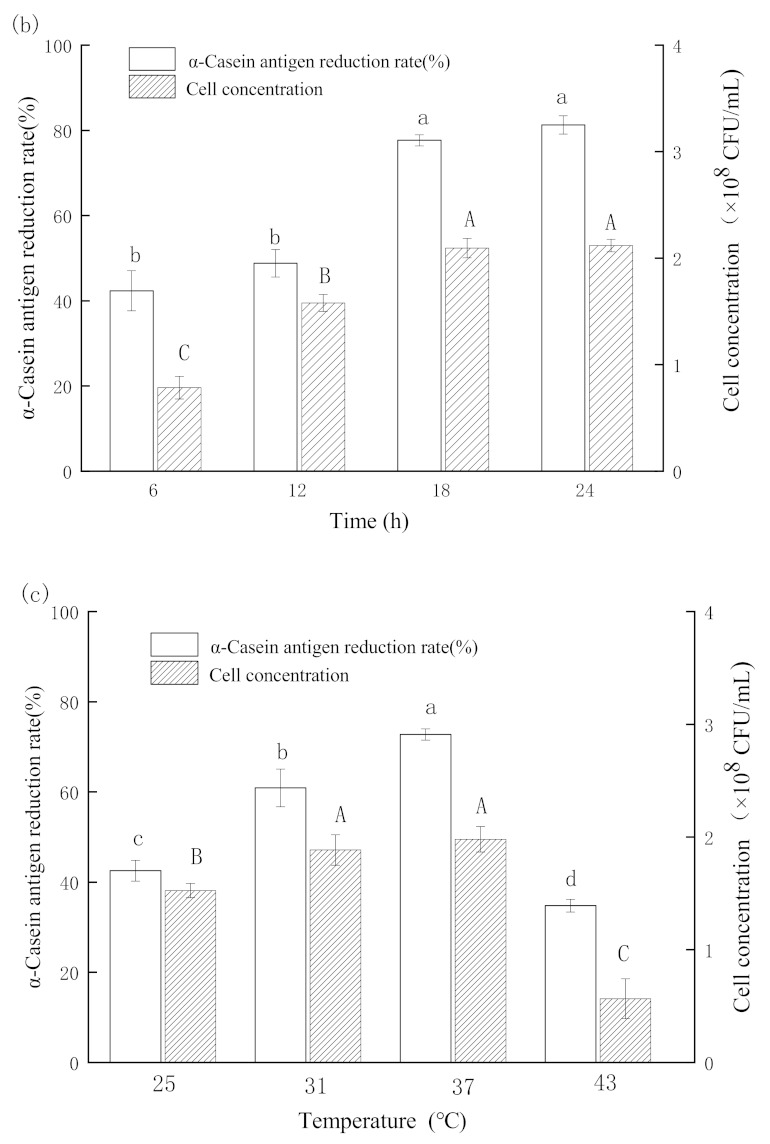
The effects of inoculation amount (**a**), fermentation time (**b**) and fermentation temperature (**c**) on the reduction rate of α-casein antigen and cell concentration of fermented milk. Bars with lowercase letter are significantly different for α-case in antigen reduction rate (*p* < 0.05) (*n* = 3). Bars with capital letter are significantly different for cell concentration (*p* < 0.05) (*n* = 3).

The effects of inoculation amount of *L. plantarum* 7-2, fermentation temperature and fermentation time on the cell concentration, and the change rate of α-CN antigen in fermented skim milk were studied (Figure 4a–c). No significant differences were observed in the concentration of α-CN antigen when the inoculation amount of *L. plantarum* 7-2 ranged from 4% to 8%. The cell concentration of *L. plantarum* 7-2 increased with the increase in inoculation amount from 2% to 4% and then decreased from 6%, while the cell concentration did not significantly differ (*p* < 0.05) when the inoculation amount was 4% or 6%. Therefore 4% was selected as the optimal inoculation amount for further optimizing fermentation time and temperature. It was found that the decrease in α-CN antigen and cell concentration of fermented milk increased gradually with the extension of fermentation time (6–24 h). However, when the fermentation time exceeded 18 h, no significant differences (*p* < 0.05) were observed in the α-CN antigen concentration and colony concentration of fermented milk, so the fermentation time was fixed at 18 h for testing the effect of temperature. At the fermentation temperature of 37 °C, the highest reducing rate of α-CN antigen and colony concentration of fermented milk was obtained. Thus, the optimum condition for reducing α-CN antigen was 4% inoculation amount, fermented for 18 h at 37 °C. The results of the studies of Liao et al. on the fermentation conditions of *Lactobacillus German* were consistent with the results of our study [38]. However, the fermentation time of 18 h determined by this experimental study is considered long and, thus, not suitable for commercial production. This can potentially be addressed by combination with other strains for joint fermentation.

### 3.4. SDS-PAGE Analysis and Antigenicity Analysis from ELISA

**Figure 5:**
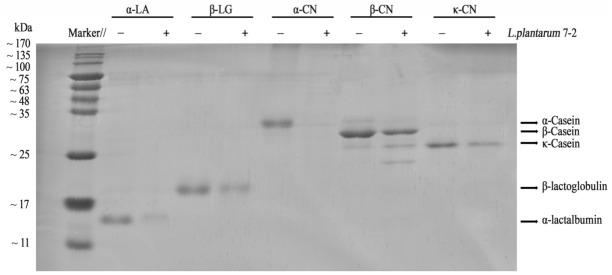
Sodium dodecyl sulfate-polyacrylamide gel electrophoresis (SDS-PAGE) of milk protein treated with *L. plantarum* 7-2 for 0 h and 18 h. Lanes marked ‘−‘ represents the standard protein of α-LA,β-LG α-CN, β-CN, and κ-CN, respectively, which have not been fermented by *L. plantarum* 7-2. ‘+’ represents the standard protein of α-LA, β-LG, α-CN, β-CN, and κ-CN, that have been fermented for 18 h by *L. plantarum* 7-2.

**Figure 6:**
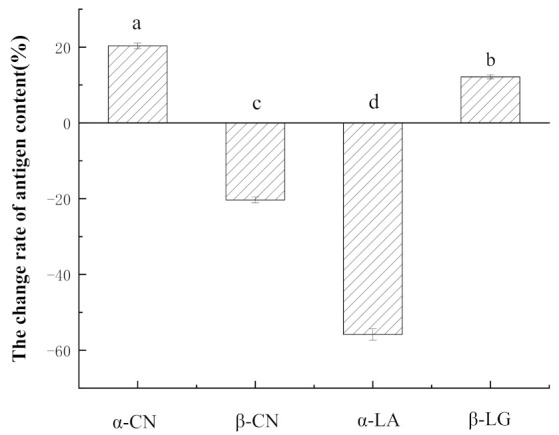
Changes in different milk protein antigens after fermentation by ELISA. Bars with lowercase letter are significantly different (*p* < 0.05) (*n* = 3).

**Figure 7:**
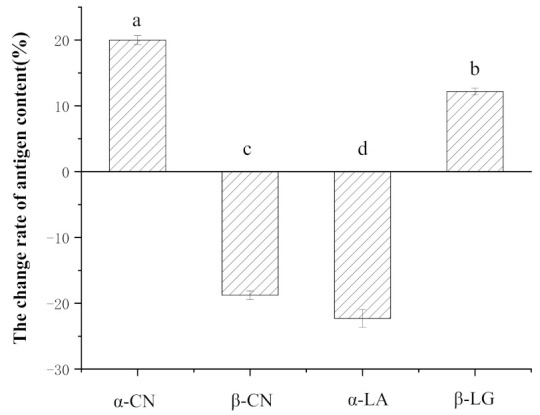
Changes in different milk protein antigens after pasteurized milk fermentation by ELISA. Bars with lowercase letter are significantly different (*p* < 0.05) (*n* = 3).

The ability of *L. plantarum* 7-2 to hydrolyze milk protein and reduce antigenicity is still unclear, as it was screened based on the amount of free amino acid and the α-CN antigenicity. *L.* *plantarum* 7-2 was cultured with α-LA, β-LG, α, β, and κ-CN to examine its ability to hydrolyze α-CN and alter the antigenicity of other milk proteins. The hydrolytic effects of *L. plantarum* 7-2 on α-LA, β-LG, α, β, and κ-CN were studied by SDS-PAGE electrophoresis (Figure 5). From the electrophoresis figure, we can see that after *L. plantarum* 7-2 fermentation hydrolysis, the α-CN band became unclear. Additionally, there was no molecular band larger than 10 kDa in that lane, indicating that α-CN was hydrolyzed into proteins or peptides smaller than 10 kDa after *L. plantarum* 7-2 fermentation, which is likely to destroy the allergic epitopes and reduce antigenicity. The antigen concentration was determined to have decreased by 20.33% (Figure 6). There are slight bands of κ-CN and β-LG cannot be adequately degraded during *L. plantarum* 7-2 fermentation and the concentration of β-LG antigen decreased by 12.15%. The band of α-LA content could not be distinguished, indicating that most of the α-LA was hydrolyzed into protein segments or peptides smaller than 10 kDa after 18 h of fermentation but its antigen concentration increased by 55.84%. Similarly, after *L. plantarum* 7-2 fermentation, β-CN showed obvious bands and produced two protein bands of mass lower than the molecular weight of the full-length protein, which may be due to the cleavage of β-CN protein chains under the action of *L. plantarum* 7-2 protease during the fermentation process, resulting in two peptides of the macromolecular proteins. On the contrary, the antigen concentration of β-CN increased by 20.33%. A similar trend can be found in fermented pasteurized milk with *L. plantarum* 7-2 (Figure 7). It was found that the antigenicity of other proteins except for α-LA basically did not change, and the antigenicity change of α-LA may be caused by the more intense sterilization conditions of reconstituted milk than pasteurization.

The increase of α-LA and β-CN may be due to the exposure of allergic epitopes of α-LA and β-CN due to hydrolysis after *L. plantarum* 7-2 fermentation. Therefore, it is not positively correlated between hydrolyzation degree and the reduction of milk protein allergens through LAB fermentation. This is a reminder that the ability of *Lactobacillus casei* to hydrolyze α-CN is superior in other LAB [39]. However, there is not enough evidence to prove that *Lactobacillus casei* is stronger in reducing protein allergenicity than other LAB, as far as we know, especially considering the strain specificity. Although this article provides a faster method of screening antigenicity reducing LAB, *L. plantarum* 7-2 is still not ideal enough because the increases the antigenicity of α-LA and β-CN, which can be resolved by mixing with other antigenicity reducing LAB to develop a compound starter.

## 4. Conclusions

In this study, a strain of *L. plantarum* 7-2 was screened from Xinjiang traditional fermented milk. It was found that *L. plantarum* 7-2 played a positive role in reducing α-CN and β-LG antigenicity which are the main allergens in bovine milk. This shows that *L. plantarum* 7-2 can effectively reduce the allergy to dairy products.

LAB is a kind of bacteria with good fermentation performance and is edible and safe. In recent years, researchers pay more and more attention to the application of LAB in reducing the allergy of dairy products, but the mechanism of LAB in reducing the allergy of milk protein is not clear. Although most reports believe that the extracellular protease system of LAB plays a role: milk protein is hydrolyzed by extracellular protease, which destroys the allergic epitope of milk protein and plays a role in desensitization. However, there are still reports that the metabolites of LAB and immune function can affect milk protein allergy. In addition, LAB fermentation desensitization is affected by fermentation time, temperature, and strain specificity, so it is necessary to study the conditions of LAB fermentation to reduce the allergy of dairy products. Due to the specificity of *Lactobacillus* hydrolyzed milk protein, combined fermentation with a variety of desensitized strains may be an important means of *Lactobacillus* desensitization.

## Figures and Tables

**Table 1:** Content of biogenic amines in dairy products fermented by *L. plantarum* 7-2 Biogenic amine content.

Biogenic Amine	Content (mg/kg)
Tryptamine	4.2345 ± 0.0715 ^a^
Putrescine	0 ± 0.0303 ^d^
Cadaverine	2.4258 ± 0.0408 ^b^
Histamine	0.5827 ± 0.3727 ^c^
Tyramine	2.9750 ± 0.005 ^b^
Spermidine	3.7835 ± 0.0725 ^a^

Bars with lowercase letter are significantly different (*p* < 0.05) (*n* = 3).

**Table 2:** Drug resistance of *L. plantarum* 7-2.

Antibiotic	Diameter of Bacteriostatic Zone/mm	Sensitivity
Ampicillin	38.16 ± 0.01 ^b^	H
Clindamycin	40.02 ± 0.01 ^a^	H
Tetracycline	19.24 ± 0.79 ^e^	H
Chloramphenicol	34.03 ± 0.03 ^c^	H
Gentamicin	1.11 ± 0.17 ^f^	L
Erythromycin	23.48 ± 0.20 ^d^	H
Vancomycin	0	L

H = highly sensitive, I = moderate sensitivity, L = insensitive. Bars with lowercase letter are significantly different (*p* < 0.05) (*n* = 3).

## Data Availability

The data presented in this study are available on request from the corresponding author. The data are not publicly available due to privacy.

## References

[1] Weinberg E.G. (2011). The WAO white book on allergy 2011–2012. Curr. Allergy Clin. Immunol..

[2] Devdas J.M., Mckie C., Fox A.T., Ratageri V.H. (2018). Food Allergy in Children: An Overview. Indian J. Pediatr..

[3] Fritsché R. (2003). Role for technology in dairy allergy. Aust. J. Dairy Technol..

[4] Dupont C. (2014). Diagnosis of cow’s milk allergy in children: Determining the gold standard?. Expert Rev. Clin. Immunol..

[5] Rosario C.S., Filho N.R. (2020). Childhood Anaphylaxis: State of the Art. Curr. Treat. Options Allergy.

[6] Dunlop J.H., Keet C.A. (2018). Epidemiology of Food Allergy. Immunol. Allergy Clin. N. Am..

[7] Sophia T., Kostas D., Kostas P. (2014). Cow’s milk allergenicity. Endocr. Metab. Immune Disord. Drug Targets.

[8] Cerecedo I., Zamora J., Shreffler W., Lin J., Dieguez M., Bardina L., Wang J., De La Hoz B., Sampson H. (2008). Mapping of the IgG and IgE Sequential Epitopes of Milk Allergens Using a Peptide Microarray-Based Immunoassay. J. Allergy Clin. Immunol..

[9] Bu G., Luo Y., Chen F., Liu K., Zhu T. (2013). Milk processing as a tool to reduce cow’s milk allergenicity: A mini-review. Dairy Sci. Technol..

[10] Lovegrove J.A., Osman D.L., Morgan J.B., Hampton S.M. (1993). Transfer of cow’s milk beta-lactoglobulin to human serum after a milk load: A pilot study. Gut.

[11] Syed Q.A., Hassan A., Sharif S., Ishaq A., Saeed F., Afzaal M., Hussain M., Anjum F.M. (2021). Structural and functional properties of milk proteins as affected by heating, high pressure, Gamma and ultraviolet irradiation: A review. Int. J. Food Prop..

[12] Eigenmann P.A., Frossard C.P. (2003). The T lymphocyte in food-allergy disorders. Curr. Opin. Allergy Clin. Immunol..

[13] Byun M.-W., Kim J.-H., Lee J.-W., Park J.-W., Hong C.-S., Kang I.-J. (2000). Effects of Gamma Radiation on the Conformational and Antigenic Properties of a Heat-Stable Major Allergen in Brown Shrimp. J. Food Prot..

[14] Kleber N., Hinrichs J. (2007). Antigenic response of β-lactoglobulin in thermally treated bovine skim milk and sweet whey. Milchwissenschaft.

[15] Peñas E., Préstamo G., Polo F., Gomez R. (2006). Enzymatic proteolysis, under high pressure of soybean whey: Analysis of peptides and the allergen Gly m 1 in the hydrolysates. Food Chem..

[16] Pintado M.E., Malcata F.X. (2000). Hydrolysis of ovine, caprine and bovine whey proteins by trypsin and pepsin. Bioprocess Eng..

[17] Kim S.B., Ki K.S., Khan M.A., Lee W.S., Lee H.J., Ahn B.S., Kim H.S. (2007). Peptic and Tryptic Hydrolysis of Native and Heated Whey Protein to Reduce Its Antigenicity. J. Dairy Sci..

[18] Hussein S., Gelencsér É., Polgár M., Hajós G. (2000). Effect of enzymatic modification on the biological activity and nutritive value of cow and buffalo casein. Acta Aliment..

[19] Cross M., Stevenson L., Gill H. (2001). Anti-allergy properties of fermented foods: An important immunoregulatory mechanism of lactic acid bacteria?. Int. Immunopharmacol..

[20] Bu G., Luo Y., Zhang Y., Chen F. (2010). Effects of fermentation by lactic acid bacteria on the antigenicity of bovine whey proteins. J. Sci. Food Agric..

[21] Fotschki J., Szyc A., Wróblewska B. (2015). Immunoreactivity of lactic acid-treated mare’s milk after simulated digestion. J. Dairy Res..

[22] El-Ghaish S., Dalgalarrondo M., Choiset Y., Sitohy M., Ivanova I., Haertlé T., Chobert J.-M. (2010). Characterization of a new isolate of Lactobacillus fermentum IFO 3956 from Egyptian Ras cheese with proteolytic activity. Eur. Food Res. Technol..

[23] El-Ghaish S., Rabesona H., Choiset Y., Sitohy M., Haertlé T., Chobert J.-M. (2011). Proteolysis byLactobacillus fermentumIFO3956 isolated from Egyptian milk products decreases immuno-reactivity of αS1-casein. J. Dairy Res..

[24] Ying Z., Luo Y. (2010). Effect of fermentation in combination with protease hydrolysis on the antigenicity of whey protein. J. Dairy Sci. Technol..

[25] Wroblewska B., Karamac M., Amarowicz R., Szymkiewicz A., Troszynska A., Kubicka E. (2004). Immunoreactive properties of peptide fractions of cow whey milk proteins after enzymatic hydrolysis. Int. J. Food Sci. Technol..

[26] Biscola V., Tulini F., Choiset Y., Rabesona H., Ivanova I., Chobert J.-M., Todorov S., Haertlé T., Franco B. (2016). Proteolytic activity of Enterococcus faecalis VB63F for reduction of allergenicity of bovine milk proteins. J. Dairy Sci..

[27] Chobert J.M., Bertrand-Harb C., Nicolas M.G., Gaertner H.F., Puigserver A.J. (1987). Solubility and emulsifying properties of caseins chemically modified by covalent attachment of L-methionine and L-valine. J. Agric. Food Chem..

[28] Shi J., Luo Y., Xiao Y., Li Z., Xu Q., Yao M. (2014). Effects of fermentation by Lactobacillus casei on the antigenicity and allergenicity of four bovine milk proteins. Int. Dairy J..

[29] Church F.C., Swaisgood H.E., Porter D.H., Catignani G.L. (1983). Spectrophotometric Assay Using o-Phthaldialdehyde for Determination of Proteolysis in Milk and Isolated Milk Proteins. J. Dairy Sci..

[30] Jorgensen J.H., Turnidge J.D. (2015). Susceptibility Test Methods: Dilution and Disk Diffusion Methods. Manual of Clinical Microbiology.

[31] Liu J., Chen L., Liu S., Qiao W., Shi R., Jiang T. (2018). Determination of biogenic amines in different dairy products byahigh performance liquid chromatography method. China Dairy Ind..

[32] Messick J.B., Berent L.M., Cooper S.K. (1998). Development and Evaluation of a PCR-Based Assay for Detection of Haemobartonella felis in Cats and Differentiation of H. felis from Related Bacteria by Restriction Fragment Length Polymorphism Analysis. J. Clin. Microbiol..

[33] Ding F.Q., Kai Q., Zhong Q.D., Lv X.L., Li J.Y., Xiong Z.H. (2012). Evaluation of available protein components in milk-based infant formula by SDS-PAGE. Food Ferment. Ind..

[34] Wu X., Liu M., Xia L., Wu H., Liu Z., Xu X. (2013). Conjugation of functional oligosaccharides reduced in vitro allergenicity of β-lactoglobulin. Food Agric. Immunol..

[35] Jedrychowski L. (1999). Reduction of the Antigenicity of Whey Proteins by Lactic Acid Fermentation. Food Agric. Immunol..

[36] Sharma C., Gulati S., Thakur N., Singh B.P., Gupta S., Kaur S., Mishra S.K., Puniya A.K., Gill J.P.S., Panwar H. (2017). Antibiotic sensitivity pattern of indigenous lactobacilli isolated from curd and human milk samples. 3 Biotech.

[37] Jin Y., Luo B., Cai J., Yang B., Zhang Y., Tian F., Ni Y. (2021). Evaluation of indigenous lactic acid bacteria of raw mare milk from pastoral areas in Xinjiang, China, for potential use in probiotic fermented dairy products. J. Dairy Sci..

[38] Liao P., Luo Y., Li Z., & Liu X. (2012). Effect of fermentation with Lactobacillus delbrueckii subsp. Bulgaricus on the anti-genicity of β-casein in cow’s milk. China Dairy Ind..

[39] El Soda M., Desmazeaud M.J., Le Bars D., Zevaco C. (1986). Cell-Wall-Associated Proteinases in Lactobacillus casei and Lactobacillus plantarum. J. Food Prot..

